# Social Support and Substance Use as Moderators of the Relationship Between Depressive Symptoms and Suicidal Ideation in Adolescents

**DOI:** 10.3389/fpsyg.2020.539165

**Published:** 2020-09-29

**Authors:** Andrés Rubio, Juan Carlos Oyanedel, Fernanda Cancino, Luna Benavente, Cristián Céspedes, Camila Zisis, Dario Páez

**Affiliations:** ^1^Facultad de Enfermería, Universidad Andres Bello, Santiago, Chile; ^2^Facultad de Psicología, Universidad Diego Portales, Santiago, Chile; ^3^Facultad de Educación y Ciencias Sociales, Universidad Andres Bello, Santiago, Chile; ^4^Centro de Investigación para la Educación Inclusiva, Viña del Mar, Chile; ^5^Facultad de Psicología y Psicopedagogía, Pontificia Universidad Católica Argentina, Buenos Aires, Argentina; ^6^Facultad de Administración y Economía, Universidad de Santiago de Chile, Santiago, Chile; ^7^Faculty of Psychology, University of the Basque Country, San Sebastian, Spain

**Keywords:** depression, suicidal ideation, social support, alcohol use, moderation, substance use

## Abstract

Literature reports that depressive symptoms may precede suicidal ideation. Several studies have identified social support and substance use as moderators of this relationship. However, no study has evaluated these variables together by testing how substance use can affect the moderating effect of social support in this relationship. The purpose of this article is to individually evaluate dimensions of social support (friends, family, significant others, and school) and substance use (alcohol, marijuana, and other illicit drugs), as moderators of the relationship between depressive symptoms and suicidal ideation, as well as analyze the moderating role of substance use in the moderation exerted by social support in this relationship. This study, quantitative and cross-sectional, considered 775 adolescents [Average age = 15.48 (*SD* = 0.96), 45.9% women], from 20 randomly selected schools in Santiago de Chile. Simple moderation models were used to analyze possible moderators separately, and double moderation models were used to analyze the moderating role of substance use in the moderating effect of social support. The results show that the four dimensions of social support moderate the relationship between depressive symptomatology and suicidal ideation, showing the strongest interaction in the case of family support, followed by support of a significant person, support at school, and support of friends, in that order. On the other hand, alcohol was the only drug that moderated the relationship in question. In addition, the results show that the use of alcohol limits the moderating effect of social support in the fields of family, significant person, and school support, but not in the case of support of friends. The use of marijuana and other illicit drugs did not affect the moderating effects of social support for any of the areas evaluated. The results are discussed according to the different roles that alcohol use can play in adolescence, and how these, together with perceived social support, are related to the emergence of suicidal ideation from depressive symptoms.

## Introduction

Globally, depressive disorders are the mental disorders that cause the most disability-adjusted life years (Rehm and Shield, 2019). These disorders, whose prevalence has its peak in adolescence (Patel, 2013; Whiteford et al., 2013), have serious consequences in various areas of people’s lives. In the particular case of adolescents, evidence shows that depressive disorders are related to problems in areas as varied as general health, other mental health problems, interpersonal relationships, and their educational/work trajectories, among others, both in adolescence itself and in future adult life (Fergusson and Woodward, 2002; Keenan-Miller et al., 2007; McLeod et al., 2012, 2016; Johnson et al., 2018).

The most relevant of this disorder group is major depression. The diagnostic criteria from the Diagnostic and Statistical Manual of Mental Disorders (DSM-V) define the major depressive disorder based on the presence of a series of symptoms that “cause clinically significant distress or impairment in social, occupational, or other important areas of functioning” (American Psychiatric Association [APA], 2013, p. 161). The nine symptoms considered are: depressed mood, loss of interest or pleasure, appetite change, sleep disturbance, psychomotor changes, decreased energy, sense of worthlessness, impaired ability to think, concentrate, or make even minor decisions, and thoughts of death, suicidal ideation, or suicide attempts. Among these, at least five must occur during a period of 2 weeks, and at least one of them must be (a) depressed mood or (b) loss of interest.

The three elements considered in the suicidal symptoms usually appear gradually, starting from thoughts of death, to suicidal ideation, until ultimately reaching suicide attempt and committing suicide (Gómez et al., 2018). The mortality associated with this disorder, which is considered high, is explained almost entirely by consummated suicide (American Psychiatric Association [APA], 2013).

However, not all depressions have suicidal symptoms. In addition, when these symptoms show, they usually do so in the last instance and can be explained as the consequence of all the others (Gómez et al., 2018; Quintana-Orts and Rey, 2018). In this sense, only in some cases of depression, non-suicidal depressive symptoms precede suicidal depressive symptoms. Considering this, several authors have paid attention to variables that facilitate or prevent this step, in order to identify intervention points. Two examples of these variables are social support and substance use.

Several studies conducted in the adolescent population have reported a negative relationship between depression and perceived social support from the family (Rojas et al., 2012), peers/friends (Resset, 2016), school (Hernández, 2017), and other significant people (Lara et al., 2004; Erhardt and Lu, 2016). The same applies to the relationship between perceived social support and thoughts of death, suicidal ideation, or suicide attempts (Salvo Garrido and Melipillán, 2008; Sarmiento et al., 2010; Andrade, 2012; Morales et al., 2017). Other research has linked these three variables, concluding that perceived social support (from different actors) moderates the relationship between depression and suicidal symptoms (the more social support, the weaker the relationship), being the most important moderating effect in the case of family support (Brausch and Decker, 2014; Lamis et al., 2016; Fredrick et al., 2018).

Otherwise, there is also evidence on the relationship between substance use, depression, and suicidal symptoms in the adolescent population. Several studies have shown evidence of the positive relationship between alcohol consumption and depressive symptoms (Marmorstein, 2009; Anseán, 2014), as well as thoughts of death, suicidal ideation, or suicide attempts (Sanchis and Domènech, 2012; Bobes et al., 2017). There are also studies that show evidence of the relationship between depressive symptoms and use of marijuana and other illegal drugs (Pardo et al., 2004; Hallfors et al., 2005; Rojas et al., 2012), and there are other studies that show evidence that the use of illegal drugs is a risk factor for suicide in young people with depressive symptoms (Hallfors et al., 2004; Conner et al., 2017). In particular Dvorak et al. (2013), show specific evidence that alcohol consumption moderates the relationship between depression and suicidal ideation: the higher the consumption, the stronger the relationship between depression and suicidal ideation.

As shown above, evidence supports the moderating effect of perceived social support and substance use (at least in the case of alcohol) in the relationship between depression and suicidal symptoms. However, there are no studies that have evaluated the interaction of these moderating effects. It is necessary to investigate how these variables interact, considering that the capacity of social support to prevent suicide in adolescents with depressive symptoms could be impaired by substance use.

Taking this into account, it is necessary to ask, How does the use of substances moderate the moderating effect of social support in the relationship between depressive symptoms and suicidal ideation? In this sense, the present study had, as its first objective, to analyze how different types of social support (from family, friends, a significant person, and school) and substance use (alcohol, marijuana, and other illegal drugs) moderate the relationship between depressive symptoms and suicidal ideation in adolescent population. As a second objective, it is sought to analyze the moderating effect of substance use on the moderating effect of social support in the relationship between depressive symptoms and suicidal ideation.

## Materials and Methods

### Participants

This study, quantitative and cross-sectional, considered 775 students in their first and second years of high school in the Chilean educational system, in the urban area of the Metropolitan Region of Chile. The average age was 15.48 (*SD* = 0.96), 45.90% female. The sampling method consisted of a probabilistic and two-stage design, where the first-level units were schools and the final-level units were the first and second years of secondary school. The sampling frame was composed of 2,484 schools belonging to Santiago (Center, South, North, East, and West), obtained from the official list of the Ministry of Education of Chile for 2017 (Ministerio de Educación, n.d.). The schools were selected using a random number generator that assigns a number for each school, while the classes within the schools were selected using a Kish selection grid (in cases where there was more than one class per level in the school).

### Measures

#### Youth’s Inventory-4

The Youth’s Inventory-4 (YI-4) questionnaire (Gadow et al., 2002), was intended to evaluate mental health in adolescents whose age fluctuated from 12 to 18 years. This instrument aims at determining the risk of emotional and behavioral disorders via closed self-report questions. It considers a set of 120 items regarding symptoms, assessed on a Likert scale from 0 to 3 points. It examines 18 categories in consonance with the frequency they are reported (0, never; 1, sometimes; 2, often; 3, very often). The items of each dimension were designed by the diagnostic criteria of the DSM-IV. Regarding psychometric properties, the authors reported good reliability and discriminant validity (according with clinical diagnoses). For this article, only the items of depression subscale and the items referring to substance use were considered. The depression subscale is made up of eight items: “I don’t feel happy, I feel sad,” “I don’t feel like doing anything,” “I think about death or suicide,” “I don’t like how I am,” “I feel tired, I have no energy to do things,” “I eat a lot,” “I sleep a lot,” and “I have a hard time concentrating.” Based on what was theoretically argued in the introduction of the article, the depression dimension for this study was constructed by adding a score obtained in each item of the subscale depression, excluding the item “I think about death or suicide,” which was considered as a separate dependent variable of the study. In the original study (Gadow et al., 2002), a Cronbach’s alpha of 0.82 was reported for the depression dimension (eight items). In this study, the depression dimension, made up of seven items, shows acceptable reliability (Cronbach’s alpha = 0.74). The items referring to substance use were “I drink alcoholic beverages (beer, wine, and other spirits),” “I smoke marijuana,” and “I use other illegal drugs (cocaine, crack, LSD, and ecstasy, etc.).”

#### Scale of Perceived Social Support in Adolescent Population

The scale of perceived social support, originally developed by Zimet et al. (1988) and validated for the adolescent population in the Chilean context by Mosqueda Díaz et al. (2015), aims to measure perceived social support in three dimensions: family, friends, and another significant person. It has 12 items (four for each dimension), which consist of statements about perceived social support that must be evaluated on a Likert scale with values from one to five (1, strongly disagree; 5, strongly agree).

The dimension support from the family is made up of the following items “My family really tries to help me,” “I get the emotional help and support I need from my family,” “I can talk about my problems with my family,” and “My family is willing to help me make decisions.” Both in the Chilean validation of the instrument and in this study, this group of items showed good reliability (Cronbach’s alpha = 0.85 and 0.87, respectively). In the case of support from friends, the dimension considered the items “My friends really try to help me,” “I can count on my Friends when things go wrong,” “I have friends with whom I can share my joys and sorrows,” and “I can talk about my problems with my friends.” Excellent reliability was observed (Cronbach’s alpha = 0.92) and is similar to that reported in the Chilean validation of the scale (Cronbach’s alpha = 0.89). The dimension of social support from a significant person consisted of: “There is a special person who is around when I need them,” “There is a special person with whom I can share my joys and sorrows,” “I have a special person who is a real source of comfort for me,” and “There is a special person in my life who cares about my feelings.” It achieved the same reliability as the validation of the scale for the Chilean context, which is acceptable (Cronbach’s alpha = 0.79).

In order to also consider a crucial dimension for adolescents, a fourth dimension of perceived social support was included in this study, referring to the perceived support at school, considering the following items: “I feel supported by my head teacher,” “I feel supported by the principal of my school,” and “I feel supported by my classmates.” These four items together showed good reliability (Cronbach’s alpha = 0.80).

The scores of each dimension were calculated as the average of the four items that make them up.

### Data Analysis

First, descriptive analyses of the study variables were performed (calculation of minimum and maximum scores, means and standard deviations, and frequencies, in the case of items of substance use and thoughts of death/suicidal ideation). A bivariate correlation analysis (Spearman’s rho) was then performed among all the variables, in order to observe how they were associated.

Subsequently, seven simple moderation analyses were performed, considering depression as an independent variable and suicidal ideation as a dependent variable, in all models, and using the four types of social support (family, friends, another significant person, and school) and the three types of substance uses (alcohol, marijuana, and other illicit drugs) as moderators. Moderation analyses allow us to analyze whether the relationship between two variables is affected by the values of a third variable (Hayes, 2018). In this case, they will allow us to analyze whether the relationship between depression and suicidal ideation varies according to the different levels of perceived social support or substance use by adolescents.

Finally, a double moderation analysis was carried out in order to test how the substance uses that were statistically significant in the simple models moderated the moderating effect of the different types of social support in the relationship between depression and suicidal ideation. Due to the significant differences by sex and age observed in adolescents in depression and substance use reported in the previous literature (Eisenberg et al., 2014; Salk et al., 2017), all moderation analyses included sex and age as control variables. The statistical analyses were carried out through the IBM-SPSS v.24 program and the modeling tool PROCESS for SPSS v2.10 (Hayes, 2018).

### Ethical Considerations

The present study has been approved by the ethics committee of the Faculty of Education, Andrés Bello University according to resolution 15/2017 of the year 2017.

## Results

### Descriptive Results

Table 1 shows the minimum and maximum scores, mean, and the standard deviation of each of the variables considered in the study.

**TABLE 1:** Descriptive analysis of the study variables.

	Minimum	Maximum	*M*	*SD*
Thoughts of death or suicidal ideation	0.00	3.00	0.52	0.85
Depression	0.00	21.00	8.65	4.11
Family perceived support	1.00	5.00	3.71	0.98
Friends perceived support	1.00	5.00	3.71	1.03
Significant person perceived support	1.00	5.00	3.83	0.90
School perceived support	1.00	5.00	3.07	0.93
Alcohol use	0.00	3.00	0.51	0.70
Marijuana use	0.00	3.00	0.30	0.71
Other illegal drug use	0.00	3.00	0.06	0.35

Table 2 shows the frequencies that participants declared about presenting thoughts of death or suicidal ideation and using alcohol, marijuana, and other illegal drugs. As shown, 65.81% of the participants stated that they had never had thoughts of death or suicidal ideation. Moreover, 40.65% of the participants declare to consume alcohol (with different frequency levels), while this percentage reaches 18.06% for the use of marijuana and 3.23% for the use of other illegal drugs.

**TABLE 2:** Frequency of substance use and thoughts of death or suicidal ideation.

	Thoughts of death or suicidal ideation		Alcohol use		Marijuana use		Other illegal drug use	
	*n*	%	*n*	%	*n*	%	*n*	%
Never	510	65.81	460	59.35	635	81.94	750	96.77
Often	176	22.71	254	32.77	78	10.06	15	1.94
Very often	42	5.42	45	5.81	35	4.52	2	0.26
Always	47	6.06	16	2.06	27	3.48	8	1.03

### Correlations

Table 3 presents the Spearman correlation coefficients (Spearman rho), among all the variables. It can be seen that both thoughts of death or suicidal ideation and depression were associated negatively with the four types of perceived social support and positively with the three types of substance use. All these associations were statistically significant (*p* < 0.01). In addition, the three types of substance use evaluated showed a negative and statistically significant relationship (*p* < 0.01) with perceived family support. This did not occur for the other types of perceived social support evaluated, which showed no association with substance use.

**TABLE 3:** Bivariate correlation matrix (Spearman’s rho) for the study variables.

	1	2	3	4	5	6	7	8	9	10	11
1. Thoughts of death or suicidal ideation	1										
2. Depression	0.41**	1									
3. Family perceived support	−0.35**	−0.31**	1								
4. Friends perceived support	−0.16**	−0.11**	0.31**	1							
5. Significant person perceived support	−0.17**	−0.14**	0.47**	0.60**	1						
6. School perceived support	−0.17**	−0.20**	0.37**	0.43**	0.33**	1					
7. Alcohol use	0.15**	0.20**	−0.17**	0.06	0.03	–0.06	1				
8. Marijuana use	0.15**	0.11**	−0.19**	0.06	–0.05	–0.04	0.45**	1			
9. Other illegal drug use	0.13**	0.10**	−0.11**	0.01	0.03	–0.06	0.27**	0.35**	1		
10. Sex (0, male; 1, female)	0.13**	0.33**	−0.10**	0.01	0.03	−0.12**	0.10**	–0.01	–0.07	1	
11. Age (in years)	0.01	–0.01	–0.04	0.03	0.02	0.00	0.13**	0.15**	0.15**	0.02	1

**The correlation is significant at the 0.01 level (bilateral).

### Simple Moderation Analysis

In this section, the results of the simple moderation analyses performed are presented. These models considered thoughts of death or suicidal ideation as the dependent variable, depression as an independent variable, and the different types of perceived social support and use of substances as moderating variables. A BCa bootstrapped CI based on 5,000 samples was used to calculate the confidence intervals of all the models used. The mean, low, and high values of the moderating variables considered their mean plus/minus a standard deviation.

To simplify the presentation of the results, the acronym TD or SI was used to replace the name of the variable *thoughts of death or suicidal ideation.*

#### Perceived Family Support as a Moderator of the Relationship Between Depression and Thoughts of Death or Suicidal Ideation

Table 4 shows the results of the linear regression model that considers TD or SI as a dependent variable and depression, perceived family support, and the interaction between them, as independent variables.

**TABLE 4:** Linear model of predictors of thoughts of death or suicidal ideation (TD or SI), considering perceived family support (*R*^2^ = 28.62%, *p* < 0.001).

	*b*	95% CI	*SE B*	*t*	*P*
Constant	−0.17	[−1.16, 0.81]	0.50	−0.34	0.73
Perceived family support	0.08	[−0.05, 0.21]	0.06	1.25	0.21
Depression	0.19	[0.15, 0.24]	0.02	8.41	<0.001
Perceived family support × Depression	−0.03	[−0.05, −0.02]	0.01	−5.44	<0.001
Sex	−0.07	[−0.18, 0.04]	0.05	−1.30	0.20
Age	−0.02	[−0.07, 0.04]	0.03	−0.55	0.58

**FIGURE 1:**
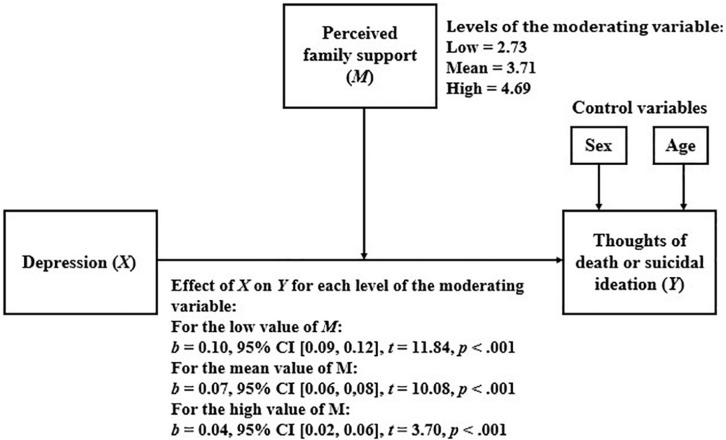
Simple moderation model considering perceived family support as moderator.

The fact that the interaction between independent variables was statistically significant for the model means that the moderation is also significant. Then, we proceeded to analyze how the relationship between depression and TD or SI varied for the different levels of perceived family support. The results of this analysis are presented in Figure 1. As shown, as perceived family support increases, the relationship between depression and TD or SI becomes weaker.

#### Perceived Friends Support as a Moderator of the Relationship Between Depression and Thoughts of Death or Suicidal Ideation

Table 5 shows the results of the linear regression model that considers TD or SI as a dependent variable and depression, perceived friends support, and the interaction between them, as independent variables.

**TABLE 5:** Linear model of predictors of TD or SI, considering perceived friends support (*R*^2^ = 20.96%, *p* < 0.001).

	*B*	95% CI	*SE B*	*t*	*p*
Constant	−0.27	[−1.26, 0.71]	0.50	−0.55	0.58
Perceived friends support	0.02	[−0.10, 0.14]	0.06	0.38	0.70
Depression	0.13	[0.09, 0.17]	0.02	6.11	<0.001
Perceived friends support × Depression	−0.01	[−0.02, 0.00]	0.01	−2.03	<0.05
Sex	−0.07	[−0.18, 0.05]	0.06	−1.13	0.26
Age	0.00	[−0.06, 0.05]	0.03	−0.06	0.95

**FIGURE 2:**
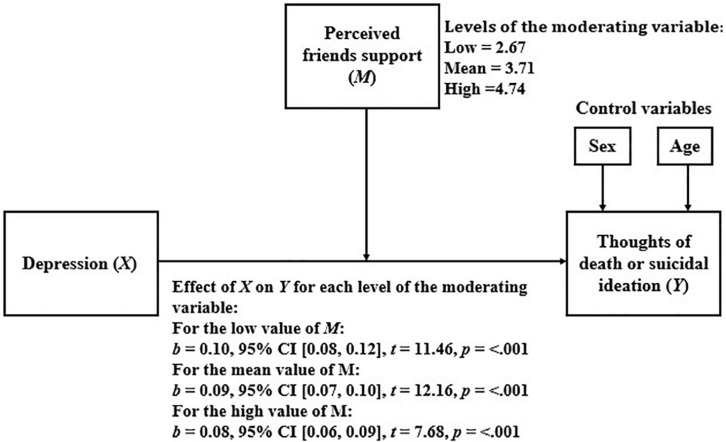
Simple moderation model considering perceived friends support as moderator.

Because the moderation was statistically significant, we proceeded to analyze how the relationship between depression and TD or SI varied for the different levels of perceived friends support. The results of this analysis are presented in Figure 2. As shown, as perceived friends support increases, the relationship between depression and TD or SI becomes weaker.

#### Significant Person Perceived Support as a Moderator of the Relationship Between Depression and Thoughts of Death or Suicidal Ideation

Table 6 shows the results of the linear regression model that considers TD or SI as a dependent variable and depression, significant person perceived support, and the interaction between them, as independent variables.

**TABLE 6:** Linear model of predictors of TD or SI, considering significant person perceived support (*R*^2^ = 22.33%, *p* < 0.001).

	*b*	95% CI	*SE B*	*t*	*p*
Constant	−0.36	[−1.38, 0.65]	0.52	−0.70	0.48
Significant person perceived support	0.06	[−0.08, 0.20]	0.07	0.84	0.40
Depression	0.17	[0.11, 0.22]	0.03	6.16	< 0.001
Significant person perceived support × Depression	−0.02	[−0.03, −0.01]	0.01	−3.02	< 0.001
Sex	−0.05	[−0.17, 0.06]	0.06	−0.94	0.35
Age	0.00	[−0.06, 0.05]	0.03	−0.17	0.86

**FIGURE 3:**
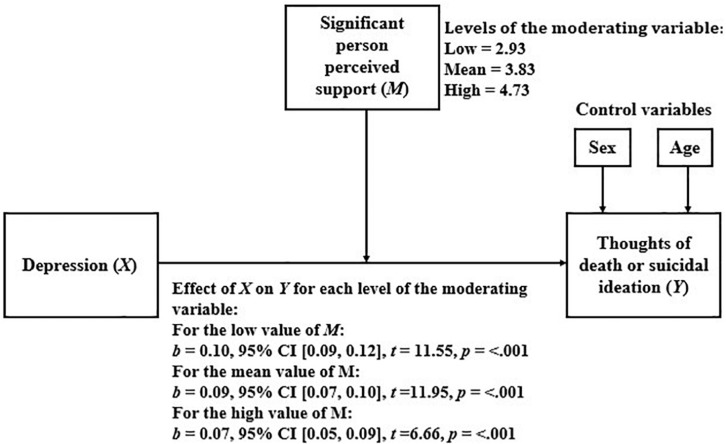
Simple moderation model considering significant person perceived support as moderator.

Because the moderation was statistically significant, we proceeded to analyze how the relationship between depression and TD or SI varied for the different levels of significant person perceived support. The results of this analysis are presented in Figure 3. As shown, as significant person perceived support increases, the relationship between depression and TD or SI becomes weaker.

#### Perceived School Support as a Moderator of the Relationship Between Depression and Thoughts of Death or Suicidal Ideation

Table 7 shows the results of the linear regression model that considers TD or SI as a dependent variable and depression, perceived school support, and the interaction between them, as independent variables.

**TABLE 7:** Linear model of predictors of TD or SI, considering perceived school support (*R*^2^ = 20.85%, *p* < 0.001).

	*b*	95% CI	*SE B*	*t*	*p*
Constant	−0.24	[−1.21, 0.73]	0.49	−0.49	0.62
Perceived school support	0.02	[−0.11, 0.15]	0.07	0.35	0.73
Depression	0.13	[0.09, 0.17]	0.02	6.34	<0.001
Perceived school support × Depression	−0.01	[−0.03, 0.00]	0.01	−2.04	<0.05
Sex	−0.08	[−0.20, 0.03]	0.06	−1.43	0.15
Age	0.00	[−0.06, 0.05]	0.03	−0.08	0.94

**FIGURE 4:**
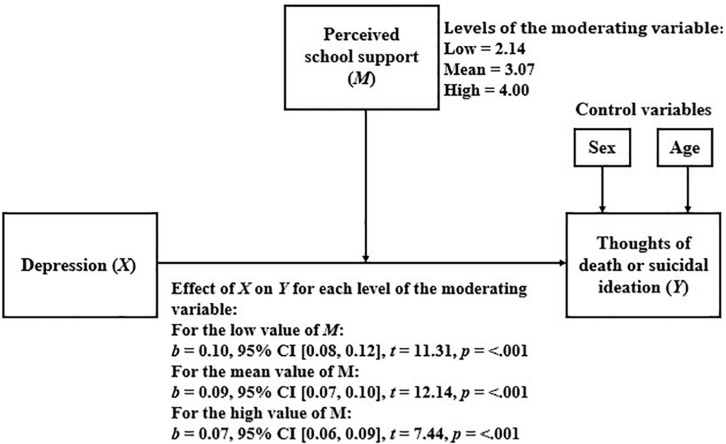
Simple moderation model considering perceived school support as moderator.

Because the moderation was statistically significant, we proceeded to analyze how the relationship between depression and TD or SI varied for the different levels of perceived school support. The results of this analysis are presented in Figure 4. As shown, as perceived school support increases, the relationship between depression and TD or SI becomes weaker.

#### Alcohol Use as a Moderator of the Relationship Between Depression and Thoughts of Death or Suicidal Ideation

Table 8 shows the results of the linear regression model that considers TD or SI as a dependent variable and depression, alcohol use, and the interaction between them, as independent variables.

**TABLE 8:** Linear model of predictors of TD or SI, considering alcohol use (*R*^2^ = 21.78%, *p* < 0.001).

	*b*	95% CI	*SE B*	*t*	*p*
Constant	0.07	[−0.82, 0.95]	0.45	0.15	0.88
Alcohol use	−0.09	[−0.28, 0.10]	0.10	−0.94	0.35
Depression	0.07	[0.06, 0.09]	0.01	8.52	<0.001
Alcohol use × Depression	0.02	[0.01, 0.04]	0.01	2.83	<0.001
Sex	−0.07	[−0.18, 0.05]	0.06	−1.14	0.25
Age	−0.02	[−0.07, 0.04]	0.03	−0.52	0.60

**FIGURE 5:**
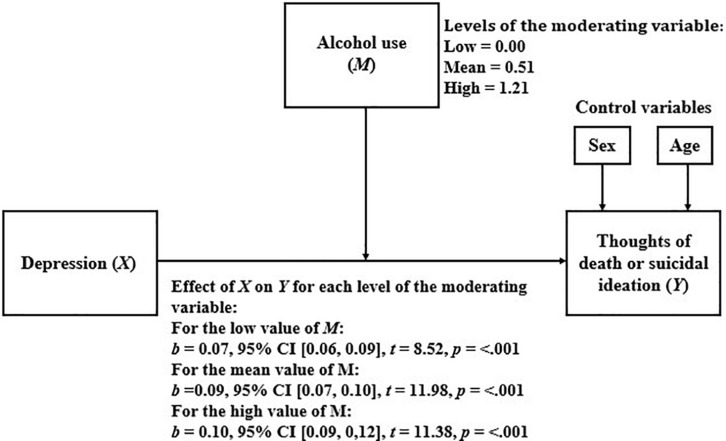
Simple moderation model considering alcohol use as moderator.

Because the moderation was statistically significant, we proceeded to analyze how the relationship between depression and TD or SI varied for the different levels of alcohol use. The results of this analysis are presented in Figure 5. As shown, as alcohol use increases, the relationship between depression and TD or SI becomes stronger.

#### Marijuana Use Support as a Moderator of the Relationship Between Depression and Thoughts of Death or Suicidal Ideation

Table 9 shows the results of the linear regression model that considers TD or SI as a dependent variable and depression, marijuana use, and the interaction between them, as independent variables.

**TABLE 9:** Linear model of predictors of TD or SI, considering marijuana use (*R*^2^ = 21.51%, *p* < 0.001).

	*b*	95% CI	*SE B*	*t*	*P*
Constant	0.13	[−0.76, 1.01]	0.45	0.28	0.78
Marijuana use	0.14	[−0.05, 0.32]	0.09	1.43	0.15
Depression	0.09	[0.07, 0.10]	0.01	11.59	<0.001
Marijuana use × Depression	0.00	[−0.01, 0.02]	0.01	0.47	0.64
Sex	−0.05	[−0.16, 0.06]	0.06	−0.86	0.39
Age	−0.03	[−0.08, 0.03]	0.03	−0.89	0.38

In this case, the interaction was not statistically significant. That is, there is no evidence to argue that the use of marijuana moderates the relationship between depression and thoughts of death or suicidal ideation.

#### Other Illegal Drug Use as a Moderator of the Relationship Between Depression and Thoughts of Death or Suicidal Ideation

Table 10 shows the results of the linear regression model that considers TD or SI as a dependent variable and depression, other illegal drug use, and the interaction between them, as independent variables.

**TABLE 10:** Linear model of predictors of TD or SI, considering other illegal drug use (*R*^2^ = 20.51%, *p* < 0.001).

	*b*	95% CI	*SE B*	*t*	*p*
Constant	−0.03	[−0.92,0.86]	0.45	−0.07	0.94
Other illegal drug use	0.62	[0.14,1.09]	0.24	2.55	<0.05
Depression	0.09	[0.08,0.11]	0.01	12.95	<0.001
Other illegal drug use × Depression	−0.03	[−0.07,0.00]	0.02	−1.72	0.09
Sex	−0.05	[−0.17,0.06]	0.06	−0.94	0.35
Age	−0.02	[−0.07,0.04]	0.03	−0.53	0.60

In this case, the interaction was not statistically significant. That is, there is no evidence to argue that the use of marijuana moderates the relationship between depression and thoughts of death or suicidal ideation.

### Double Moderation Analysis

**FIGURE 6:**
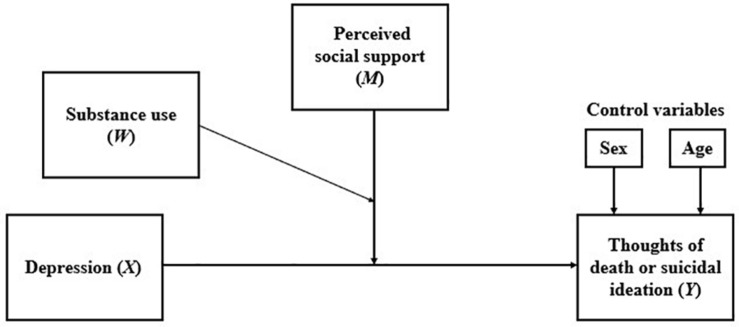
Conceptual model of double moderation analysis.

The results of the double moderation analyses are presented below, which were performed to observe how substance use can moderate the moderation of perceived social support in the relationship between depression and thoughts of death or suicidal ideation (see Figure 6).

**FIGURE 7:**
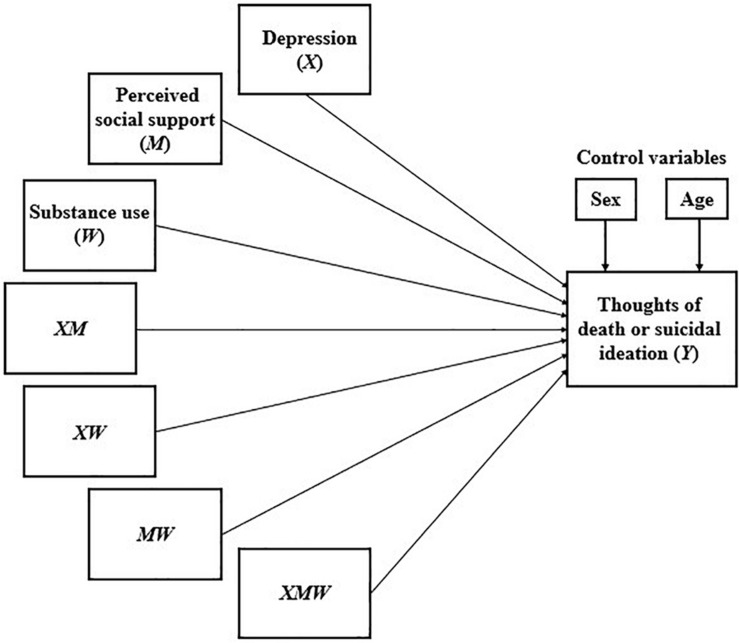
Multiple linear regression analysis for the double moderation model.

This procedure considers a multiple linear regression analysis, which includes, as a dependent variable, the thoughts of death or suicidal ideation, and as independent variables depression, perceived social support, substance use, and all possible combinations between these three variables (including the triple combination), as presented in Figure 7.

As in the previous analyses, a BCa bootstrapped CI based on 5,000 samples was used to calculate the confidence intervals of all the models used. Due to the quantity and complexity of the models, only the results of the triple interaction between depression, perceived social support, and substance use (for all combinations between the types of perceived social support and substance use) are presented, since this is the only thing that can show if the double moderation is statistically significant. These results are presented in Table 11.

**TABLE 11:** Statistical significance of the interaction between depression, perceived social support, and substance use, controlling by sex and age.

	*b*	95% CI	*SE B*	*T*	*p*
Depression × Family perceived support × Alcohol use	−0.02	[−0.03, 0.00]	0.01	−1.97	<0.05
Depression × Family perceived support × Marijuana use	−0.01	[−0.03, 0.01]	0.01	−1.27	0.21
Depression × Family perceived support × Other illegal drug use	−0.01	[−0.04, 0.02]	0.02	−0.49	0.62
Depression × Friends perceived support × Alcohol use	−0.01	[−0.02, 0.01]	0.01	−0.81	0.42
Depression × Friends perceived support × Marijuana use	0.00	[−0.01, 0.02]	0.01	0.43	0.66
Depression × Friends perceived support × Other illegal drug use	−0.03	[−0.06, 0.01]	0.02	−1.67	0.10
Depression × Significant person perceived support × Alcohol use	−0.02	[−0.04, 0.00]	0.01	−2.17	<0.05
Depression × Significant person perceived support × Marijuana use	−0.01	[−0.03, 0.01]	0.01	−0.98	0.33
Depression × Significant person perceived support × Other illegal drug use	−0.10	[−0.17, −0.03]	0.04	−2.76	<0.01
Depression × Perceived school support × Alcohol use	−0.02	[−0.03, 0.00]	0.01	−1.98	<0.05
Depression × Perceived school support × Marijuana use	−0.01	[0.00, 0.03]	0.01	1.59	0.11
Depression × Perceived school support × Other illegal drug use	−0.01	[−0.05, 0.02]	0.02	−0.81	0.42

Only four of the 12 double moderations analyzed were statistically significant. As shown, alcohol is present in three of these four models. Tables 12–15 show how the variables interacted in the models that were statistically significant.

**TABLE 12:** Conditional effect of depression on TD or IS at different levels of alcohol use and perceived family support, controlling by sex and age.

Alcohol use	Family support	*b*	*SE B*	*t*	*p*	*LLCI*	*ULCI*
0.00	2.73	0.08	0.01	7.01	<0.001	0.06	0.10
0.00	3.71	0.06	0.01	7.13	<0.001	0.04	0.08
0.00	4.69	0.04	0.01	3.61	<0.001	0.02	0.06
0.51	2.73	0.09	0.01	10.06	<0.001	0.07	0.11
0.51	3.71	0.07	0.01	9.10	<0.001	0.05	0.08
0.51	4.69	0.04	0.01	3.85	<0.001	0.02	0.06
1.21	2.73	0.11	0.01	10.29	<0.001	0.09	0.13
1.21	3.71	0.07	0.01	7.59	<0.001	0.05	0.09
1.21	4.69	0.03	0.01	2.48	<0.05	0.01	0.06

**TABLE 13:** Conditional effect of depression on TD or IS at different levels of alcohol use and significant person perceived support, controlling by sex and age.

Alcohol use	Significant person support	*b*	*SE B*	*t*	*p*	*LLCI*	*ULCI*
0.00	2.93	0.07	0.01	6.85	<0.001	0.05	0.10
0.00	3.83	0.06	0.01	7.44	<0.001	0.05	0.08
0.00	4.73	0.06	0.01	4.39	<0.001	0.03	0.08
0.51	2.93	0.10	0.01	10.74	<0.001	0.08	0.12
0.51	3.83	0.08	0.01	10.73	<0.001	0.06	0.09
0.51	4.73	0.06	0.01	5.70	<0.001	0.04	0.08
1.21	2.93	0.13	0.01	9.84	<0.001	0.10	0.16
1.21	3.83	0.10	0.01	10.59	<0.001	0.08	0.11
1.21	4.73	0.06	0.01	4.93	<0.001	0.04	0.09

**TABLE 14:** Conditional effect of depression on TDorIS at different levels of other illegal drug use and significant person support, controlling by sex and age.

Other illegal drug use	Significant person support	*b*	*SE B*	*t*	*p*	*LLCI*	*ULCI*
0.00	2.93	0.10	0.01	11.09	<0.001	0.08	0.12
0.00	3.83	0.08	0.01	11.28	<0.001	0.07	0.10
0.00	4.73	0.06	0.01	6.27	<0.001	0.04	0.08
0.06	2.93	0.11	0.01	11.70	<0.001	0.09	0.13
0.06	3.83	0.08	0.01	11.76	<0.001	0.07	0.10
0.06	4.73	0.06	0.01	6.18	<0.001	0.04	0.08
0.40	2.93	0.16	0.03	6.15	<0.001	0.11	0.21
0.40	3.83	0.10	0.01	7.18	<0.001	0.08	0.13
0.40	4.73	0.05	0.01	4.29	<0.001	0.03	0.07

**TABLE 15:** Conditional effect of depression on TD or IS at different levels of alcohol use and perceived school support, controlling by sex and age.

Alcohol use	School support	*b*	*SE B*	*t*	*p*	*LLCI*	*ULCI*
0.00	2.14	0.07	0.01	6.70	<0.001	0.05	0.09
0.00	3.07	0.07	0.01	7.48	<0.001	0.05	0.08
0.00	4.00	0.06	0.01	4.97	<0.001	0.04	0.08
0.51	2.14	0.09	0.01	10.32	<0.001	0.07	0.11
0.51	3.07	0.08	0.01	10.70	<0.001	0.06	0.09
0.51	4.00	0.07	0.01	6.33	<0.001	0.05	0.09
1.21	2.14	0.12	0.01	10.33	<0.001	0.10	0.14
1.21	3.07	0.10	0.01	10.51	<0.001	0.08	0.11
1.21	4.00	0.07	0.01	5.62	<0.001	0.05	0.10

As shown, as alcohol use increases, the ability of family support to weaken the relationship between depression and thoughts of death or suicidal ideation decreases. The same goes for alcohol in the case of significant person support and school support.

In the case of the use of other illegal drugs, the same happens: the greater the use of illegal drugs, the lower the capacity of a significant person to weaken the relationship between depression and suicidal ideation.

## Discussion

The results of this study showed that all types of social support evaluated are statistically significant moderators of the relationship between depression and thoughts of death or suicidal ideation (the higher the perceived social support, the weaker the relationship between depression and thoughts of dead or suicidal ideation, with family support having the most important moderating effect), following the line of evidence previously reported (Brausch and Decker, 2014; Lamis et al., 2016; Fredrick et al., 2018). The literature has indicated that social support helps people deal with difficulties in a better way, since a positive social environment provides confirmation of social identity (Korkiamaki and Ellonen, 2008). In this way, social support contributes by positively moderating negative events or stressors (Bliese and Britt, 2001). Especially in studies of children and adolescents, social support is seen as a sample of community social capital (Korkiamaki and Ellonen, 2008).

The same happened with the use of alcohol as a moderator of this relationship, where, as the study by Dvorak et al. (2013) showed, the higher the consumption of alcohol, the stronger the relationship between depression and thoughts of death and suicidal ideation. This could be because adolescents see in the use of alcohol an exit from their negative affective states in the short term, particularly in the case of affective states related to depression (Hussong et al., 2017), which has the consequence that they do not focus on asking for help or solving problems related to their state (Dvorak et al., 2013), which finally translates (in the long term) in the increase of negative emotional states and hopelessness. On the other hand, no evidence was found to support the idea that use of marijuana or other illegal drugs moderates this relationship.

When evaluating double moderations (moderation of substance use on the moderating effect of perceived social support in the relationship between depression and thoughts of dead or suicidal ideation), results showed that the use of alcohol affected the moderating role played by perceived social support from the family, school, and another significant person. The fact that the use of alcohol impacts social support as a protective factor could be due to the fact that people who suffer from a mental disorder tend to consume alcohol to avoid what they feel in social situations (Blumenthal et al., 2016; Collins et al., 2018). In this sense, one might think that this evasive use of alcohol would not allow for the strengthening of the social identity that social development contexts usually foster.

Furthermore, the results did not show statistically significant evidence that alcohol use affected the moderating effect of perceived social support from friends. To interpret this, it is important to note that within the four areas of perceived social support evaluated (family, friends, significant person, and school), it is precisely the area of friends where the vast majority of situations in which adolescents consume alcohol occur. Considering this, it could be thought that the use of alcohol in adolescents, in addition to having negative consequences (Marshall, 2014), could also (in the particular sphere of friends) generate a space where they can share and validate themselves with peers, strengthening ties and reinforcing the sense of belonging between peers. In this way, the negative and positive aspects of alcohol use could tend to be nullified for the particular environment of friends. This would not happen in the case of the family, another significant person, and the school, where only the negative effects of alcohol use seem to be involved.

One of the limitations of this study is its methodological design (cross sectional). Longitudinal designs may allow for observing which variables that show associations appear first, which might hint at the most appropriate variables for intervention. Another limitation of this study was the way in which depressive symptoms were measured (self-report questionnaires). Although the instrument has good validity, it will never be as accurate as the clinical interviews conducted by health professionals, who may consider a greater number of variables when diagnosing. Future studies should consider these limitations in order to precisely clarify the conditions that prevent adolescents with depressive symptoms from developing suicidal behaviors, especially considering that there is not yet enough evidence that allows us to clearly understand the relationship between the variables that come into play in this phenomenon.

## Data Availability Statement

The datasets generated for this study are available on request to the corresponding author.

## Ethics Statement

The studies involving human participants were reviewed and approved by the Ethics Committee of the Faculty of Education, Andrés Bello University according to resolution 15/2017 of the year 2017. Written informed consent from the participants’ legal guardian/next of kin was not required to participate in this study in accordance with the national legislation and the institutional requirements.

## Author Contributions

AR, JO, FC, and LB were involved in the majority of the study, including planning and supervising the work, performing the measurements, processing the experimental data, performed the analysis, drafting the manuscript, and designing the figures. CC and CZ aided in the sample design and interpreting the results, and worked on the manuscript. DP was involved in supervising the work and providing a methodological review. All authors discussed the results, commented critically on the manuscript, and read and approved the accepted version.

## Conflict of Interest

The authors declare that the research was conducted in the absence of any commercial or financial relationships that could be construed as a potential conflict of interest.
